# Plasma-based strategies for systemic rejuvenation: critical perspectives on clinical translation

**DOI:** 10.1007/s11357-026-02136-8

**Published:** 2026-02-20

**Authors:** Rafal Gulej, Roland Patai, Anna Ungvari, Attila Tordai, Derek M. Huffman, Stefano Tarantini, Andriy Yabluchanskiy, Zoltan Ungvari, Anna Csiszar

**Affiliations:** 1 Vascular Cognitive Impairment, Neurodegeneration, and Healthy Brain Aging Program, Department of Neurosurgery, University of Oklahoma Health Sciences Center, Oklahoma City, OK USA; 2 Oklahoma Center for GeroScience and Healthy Brain Aging, University of Oklahoma Health Sciences Center, Oklahoma City, OK USA; 3 International Training Program in Geroscience, Health Sciences Division, Doctoral College/Institute of Preventive Medicine and Public Health, Semmelweis University, Budapest, Hungary; 4 Institute of Preventive Medicine and Public Health, Semmelweis University, Budapest, Hungary; 5 Fodor Center for Prevention and Healthy Aging, Semmelweis University, Budapest, Hungary; 6 Department of Transfusion Medicine, Semmelweis University, Budapest, Hungary; 7 Department of Molecular Pharmacology, Albert Einstein College of Medicine, Bronx, NY 10461 USA; 8 Institute for Aging Research, Albert Einstein College of Medicine, Bronx, NY USA; 9 Department of Medicine, Albert Einstein College of Medicine, Bronx, NY USA; 10 Stephenson Cancer Center, University of Oklahoma, Oklahoma City, OK USA; 11 International Training Program in Geroscience, Doctoral College/Department of Translational Medicine, Semmelweis University, Budapest, Hungary; 12 Healthy Aging Program, Institute for Translational Research, Budapest, Hungary

**Keywords:** Parabiosis, Plasma-based interventions, Systemic rejuvenation, Aging, Therapeutic plasma exchange, Translational challenges, Biomarkers of aging

## Abstract

Experimental models such as heterochronic parabiosis and heterochronic plasma transfer have profoundly advanced our understanding of systemic aging, demonstrating that circulating factors can influence brain, vascular, and immune aging through cell nonautonomous mechanisms. These preclinical models have revealed that both pro-geronic and anti-geronic signals in blood can modulate neuroinflammation, neurovascular health, and cognitive resilience. However, despite their experimental promise, the clinical translation of these findings, particularly through plasma-based interventions in humans, remains fraught with uncertainty. This review critically examines the strengths and limitations of parabiosis-based paradigms as platforms for discovery, contrasts them with early human plasma infusion studies, and evaluates the current biological, medical, and ethical challenges associated with young plasma therapies. Therapeutic plasma exchange has also emerged as a potential rejuvenation strategy, but its mechanisms, efficacy, and longterm safety remain incompletely understood. We argue that future progress in systemic rejuvenation will depend on precise, mechanistically informed interventions—rather than broad, premature applications of plasma therapies, which require further validation through rigorous scientific investigation.

## Introduction

Experimental paradigms such as heterochronic parabiosis and plasma transfer have revolutionized our understanding of how systemic factors regulate aging through cell non-autonomous mechanisms. These models have demonstrated that circulating molecules can profoundly affect cellular and molecular mechanisms of aging and impact cardiovascular, neural, and immune functions [1–3]. More recently, plasma dilution strategies, such as therapeutic plasma exchange (TPE), have emerged as an additional experimental approach to modulate the systemic milieu, with preclinical studies reporting promising rejuvenative effects on multiple organ systems [4, 5]. However, it is important to recognize that even in these controlled experimental settings, the mechanisms underlying such effects are only partially understood. For example, while plasma dilution may attenuate certain pro-geronic factors, the potential removal of protective, anti-geronic components remains poorly characterized. Furthermore, the extent to which these interventions translate to long-term functional improvements and whether they are applicable beyond carefully selected experimental conditions remains uncertain.

Overall, these experimental models primarily serve to uncover mechanistic insights, not to endorse direct clinical applications. Benefits observed in preclinical studies are often context-dependent, modest, and non-curative. Notably, in models of advanced pathology, such as Alzheimer’s disease, exposure to young plasma does not reverse hallmark features like amyloid plaque burden [6]. Accordingly, while these studies offer valuable biological insights, existing clinical trials of plasma-based interventions do not yet provide sufficient evidence for therapeutic efficacy.

The purpose of this review is to critically evaluate the translational relevance of plasma-based interventions inspired by preclinical parabiosis and plasma dilution models. While these studies have yielded transformative insights into the systemic regulation of aging and identified promising molecular targets, the leap from mechanistic discovery to clinical application remains fraught with biological, methodological, and ethical challenges [2, 7, 8]. We aim to clarify the distinction between plasma and plasma dilution approaches as research tools versus their application as therapeutic agents, emphasizing that while these models have been invaluable for identifying pro- and anti-geronic factors, neither plasma transfer nor TPE has yet met the scientific or regulatory standards required for safe and effective use in humans for anti-aging indications.

In this review, we outline the current state of evidence from both animal models and human trials, highlight key concerns around the use of plasma-based interventions in clinical settings, and explore alternative, more targeted strategies for leveraging systemic biology in the treatment of age-related disease. Ultimately, we argue for a shift in focus—from enthusiasm for unrefined plasma-based interventions [9] to rigorous mechanistic dissection, biomarker development, and the pursuit of precision-guided, ethically sound therapeutic strategies.

## Overview of preclinical studies

Over the past two decades, heterochronic parabiosis—the surgical joining of a young and aged animal to establish shared circulation—has emerged as a powerful model to study the systemic regulation of aging [10]. These experiments have demonstrated that factors present in young blood can reverse multiple age-related phenotypes in older animals, while aged blood can impair tissue function in young partners. Importantly, these findings span multiple organ systems, including the brain, skeletal muscle, liver, heart, and vasculature, supporting the concept that aging is regulated, in part, by circulating molecular cues [2, 11–22].

Follow-up studies using plasma transfer and blood exchange paradigms provided converging evidence that acellular fractions of blood alone are sufficient to mediate many of these effects. For example, the administration of young plasma to aged mice has been shown to improve hippocampal neurogenesis, enhance synaptic plasticity, and boost cognitive performance [23]. Conversely, aged plasma impairs cognitive function in young animals and increases neuroinflammation, endothelial dysfunction, and blood–brain barrier (BBB) permeability [18, 24, 25]. These findings underscore the presence of both anti-geronic (e.g., IGF-1, GDF11, soluble Klotho, PF4, TIMP2) and pro-geronic (e.g., CCL11, B2M, TNF-α, SASP factors) components in the systemic circulation [14, 16, 24, 26–30].

More recently, plasma dilution approaches, such as neutral blood exchange (NBE) and therapeutic plasma exchange in animal models, have provided additional evidence that modifying the systemic milieu can influence aging phenotypes. In rodent studies, NBE, a procedure that dilutes plasma proteins and replaces them with saline and albumin while returning cellular components, has been associated with improvements in neurogenesis, vascular function, muscle repair, and reductions in tissue inflammation and cellular senescence[4, 5]. These results suggest that reducing or diluting age-elevated circulating factors may partially restore youthful tissue function. However, it is important to emphasize that even in these controlled preclinical settings, the precise mechanisms underlying these benefits remain incompletely understood. In particular, the extent to which plasma dilution inadvertently removes beneficial, anti-geronic factors alongside harmful signals is not fully characterized. Moreover, the durability, tissue specificity, and potential trade-offs of such interventions remain areas of active investigation. Importantly, sex-based differences, often underreported or not evaluated, may significantly influence the efficacy and safety of plasma-based interventions. Preclinical studies have shown divergent immune and regenerative responses between male and female animals, and human plasma proteomics studies indicate sex-specific aging trajectories. Future research should prioritize sex-stratified analyses to inform personalized rejuvenation strategies. In addition to sex, genetic background may significantly influence outcomes, yet the extent of strain dependency in parabiosis and plasma dilution studies remains largely unexplored. Many age-related phenotypes and responses to systemic interventions in mice are known to be strain-specific [31], raising concerns about the generalizability of findings across different genetic contexts.

While these results are compelling, it is essential to recognize their context-dependent and non-curative nature. In models of early-stage aging, youthful blood components may transiently enhance function or delay decline. However, in advanced pathological states, the impact is often limited. For instance, although young plasma improves neurogenesis and reduces inflammatory signatures in aged Alzheimer’s models, it does not reduce amyloid plaque burden or reverse tau pathology [6]. This suggests that systemic interventions may be more effective as preventive or modulatory strategies, rather than curative treatments. Indeed, a small clinical trial (NCT00742417) assessed plasma exchange with 5% albumin in Alzheimer’s disease patients to evaluate safety and its effect on beta‑amyloid clearance, but.

An early clinical investigation (NCT00742417) evaluated the safety and feasibility of plasma exchange with 5% albumin replacement in patients with Alzheimer’s disease, with particular interest in whether peripheral Aβ sequestration might influence central amyloid dynamics. The study demonstrated that repeated plasma exchange was technically feasible and generally tolerated in this patient population. However, the trial was not powered to detect clinical efficacy, and no definitive improvements in cognitive outcomes or reversal of core neuropathological features were demonstrated. As such, while the study provided important proof-of-concept data regarding safety, it did not produce definitive evidence supporting reversal of core pathology in humans.

More recently, the Alzheimer’s disease Management by Albumin Replacement (AMBAR) trial (NCT01561053, 2012) [32–35] examined the effects of plasma exchange with albumin replacement in patients with mild to moderate Alzheimer’s disease. The trial yielded mixed and preliminary results [36]. While certain subgroups demonstrated modest slowing of cognitive and functional decline [37], the study did not show robust or consistent clinical benefits across the full cohort, nor did it provide evidence for reversal of core neuropathological features. CSF Aβ biomarkers did not demonstrate a clear disease-modifying effect, and the clinical significance of imaging changes remains uncertain. Several important limitations complicate interpretation, including the absence of key covariates, such as amyloid status, APOE ε4 genotype, pre-enrollment treatments, comorbidities (e.g., diabetes), and education level, in the primary and post hoc analyses [38]. These factors are known to significantly influence disease progression and interindividual variability in treatment response. Taken together, existing human trials highlight the challenges of translating promising preclinical findings into clinically meaningful outcomes. Further large-scale, rigorously controlled studies are needed before definitive conclusions can be drawn about the therapeutic potential of plasma exchange in aging and neurodegenerative disease. In terms of safety, the AMBAR trial reported a markedly higher incidence of procedure-related adverse events (AEs) in the treatment group (10.6%) compared to the sham group (0.7%). Notably, 52.6% of full therapeutic plasma exchange (TPE) procedures were performed using central venous access, which was associated with a higher rate of AEs (20.1%) relative to procedures using peripheral access (13.1%). These findings underscore the procedural risks associated with TPE, particularly in older populations, and highlight the importance of vascular access type in influencing safety outcomes. Additionally, variability in experimental design, including differences in donor-recipient age gaps, duration of exposure, genetic background of animals, and endpoint measures, makes it difficult to extrapolate generalized conclusions. Importantly, sex-based differences, often underreported, may significantly influence outcomes.

A broader translational context further underscores the need for caution when extrapolating preclinical rejuvenation paradigms to humans. Across multiple disease domains, including cancer [39], stroke, and Alzheimer’s disease, the historical success rate of translating therapeutic efficacy from rodent models to humans has been strikingly low. Systematic analyses indicate that the majority of interventions showing robust benefit in animal models fail to demonstrate efficacy in late-phase clinical trials, often due to biological complexity, disease heterogeneity, and limitations of preclinical model systems. In Alzheimer’s disease [40] and stroke [41] in particular, promising neuroprotective strategies have repeatedly succeeded in mice yet failed in humans, despite strong mechanistic rationale and reproducibility in preclinical settings. These observations do not diminish the value of animal models as tools for discovery, but they highlight the inherent risks of viewing them as predictive therapeutic platforms. Plasma-based rejuvenation strategies should therefore be interpreted within this broader translational landscape, where mechanistic insight far outpaces clinical success.

In summary, preclinical studies using parabiosis, plasma transfer, and plasma dilution have provided unprecedented insights into cell non-autonomous aging mechanisms, demonstrating that the systemic milieu plays a central role in the regulation of tissue health and repair. However, these models are exploratory tools, not therapeutic blueprints. Their true value lies in uncovering actionable molecular targets and pathways, and not in endorsing wholesale application of young plasma or plasma dilution as anti-aging treatments [42].

Importantly, while specific factors such as IGF-1 have shown putative gerotherapeutic effects in selected models, extrapolating their efficacy across diverse aging phenotypes and organ systems remains premature. Aging is a multifactorial process, and single circulating molecules may have divergent effects depending on tissue type or disease context. For example, a factor promoting regeneration in the brain or muscle might exert pro-oncogenic effects in epithelial tissues. Thus, the most promising translational pathway lies not in broad rejuvenation attempts via plasma manipulation, but in identifying and precisely targeting mechanistically defined modulators of aging in a tissue- and context-specific manner.

## Current human trials with plasma-based interventions: promise, limitations, and the need for caution

Despite compelling findings from animal models, the translation of plasma-based interventions into clinical applications remains highly preliminary. A small number of early-phase clinical studies have explored the potential therapeutic benefits of young plasma or plasma-derived fractions in aging-related conditions, particularly cognitive decline and neurodegenerative diseases [6, 43–45]. However, the quality, rigor, and interpretability of these studies vary substantially, and none have provided conclusive evidence of clinical efficacy.

One of the most frequently cited trials is the evaluation of GRF6019, a proprietary plasma fraction derived from young donors. Preliminary findings from these open-label studies suggested possible improvements in functional and cognitive endpoints in patients with mild to moderate Alzheimer’s disease [44, 45]. However, these studies focused on tolerability and safety, were small, unblinded, and lacked appropriate placebo controls, significantly limiting their scientific validity. Moreover, they did not include long-term follow-up to assess the durability of any observed effects or to evaluate potential delayed adverse outcomes.

Other plasma infusion studies, such as the PLASMA study (NCT02256306), which involved infusing young donor plasma into older individuals with Alzheimer’s disease, have similarly reported minimal cognitive improvement and modest biological effects [46]. However, these trials did not demonstrate statistically significant or clinically meaningful outcomes compared to baseline or control groups, and their small sample sizes and short duration further limit their interpretability [46].

To date, no randomized, double-blind, placebo-controlled trial has demonstrated that young plasma or its components can produce durable improvements in cognition, physical function, or validated biomarkers of biological aging. In the absence of such rigorously generated data, the clinical relevance of these interventions remains entirely speculative. Importantly, the public perception of plasma-based therapies has outpaced the science, fueled in part by commercial ventures and media coverage [9, 47, 48] that exaggerate the potential benefits while downplaying the limitations [49–51].

Further complicating the landscape is the substantial heterogeneity in trial design, participant selection, plasma preparation protocols, and outcome measures. This variability makes cross-study comparisons challenging and hampers efforts to draw meaningful conclusions about safety, efficacy, or mechanisms of action. Many trials are further limited by small sample sizes, lack of blinding, and the absence of mechanistic biomarkers that could establish a causal link between intervention and observed effects.

Taken together, these limitations underscore a key point: plasma is not a proven anti-aging therapy. While preliminary studies suggest the concept warrants further investigation, existing evidence does not justify the clinical use of plasma infusions for aging-related conditions outside of carefully controlled research settings. While the promise of systemic rejuvenation is scientifically intriguing, it must be weighed against the current limitations of the clinical evidence base, which remains preliminary, underpowered, and methodologically diverse. Before considering alternative approaches, such as plasma dilution therapies, it is essential to carefully examine the broader medical and ethical concerns that these interventions raise.

## Medical risks and complications

Plasma transfusion is a well-established medical procedure in transfusion medicine, typically used to treat coagulation disorders, severe infections (e.g., convalescent plasma), and specific immunodeficiencies [52]. However, it is far from a benign intervention, particularly in the context of elective or non-essential use in aging populations.

Among the most pressing risks is transfusion-associated circulatory overload (TACO), a common and potentially severe complication in elderly or frail individuals [53]. Even in hospital settings, TACO remains one of the leading causes of transfusion-related morbidity [54]. Plasma transfusion can also elicit febrile non-hemolytic transfusion reactions (FNHTRs) and allergic transfusion reactions (ATRs), often self-limiting but occasionally severe [55]. More serious complications include immune-mediated reactions due to undetected alloantibodies. Perhaps even more concerning in the anti-aging context are the theoretical risks of pro-thrombotic events or aberrant growth signaling arising from the poorly understood mixture of bioactive peptides, hormones, and cytokines found in plasma [56]. Age-associated declines in certain circulating factors, including growth factors, may serve protective roles, particularly against aggressive cancers. For example, neoplasms in older individuals often exhibit a slower, indolent course. A youthful systemic environment, if introduced indiscriminately, may reawaken dormant tumor growth or promote metastatic potential. These potential pro-oncogenic effects highlight the need for rigorous safety assessments and long-term monitoring in any plasma-based rejuvenation intervention. The very molecules that may confer benefits (e.g., IGF-1) can also promote unintended angiogenesis, fibrosis, or oncogenic pathways, especially in vulnerable older adults [57–59]. The risk of transfusion-transmitted infections (TTIs) remains low in regulated settings but is not zero, especially in unregulated clinics or markets where rigorous screening and pathogen inactivation may be lacking. These include viral (e.g., HIV, HBV, HCV), bacterial, and parasitic pathogens [60].

Given these risks, modern transfusion practice increasingly favors component therapy, such as administration of recombinant coagulation factors or immunoglobulins, rather than whole plasma. Component therapy allows for volume reduction, precise dosing, pathogen inactivation, and reduced immunogenicity—features that plasma transfusion lacks when used as a crude anti-aging strategy.

## Ethical and regulatory challenges

In parallel to medical risks, a host of ethical issues surrounds the growing commercial interest in plasma-based “rejuvenation clinics.” These clinics, often operating outside of FDA oversight, offer expensive plasma infusions or TPE procedures marketed with exaggerated or scientifically unsupported claims of cognitive or biological rejuvenation. Clinics offering these treatments often target vulnerable populations, and the associated procedures can cost thousands of dollars per session [61].

One major concern is donor exploitation. In regions where financial incentives are permitted, economically vulnerable individuals, especially young adults, may be pressured into frequent plasma donation. This raises significant issues of coercion, informed consent, and social equity [62–64].

Another concern is medical tourism, where patients travel to countries with less stringent regulatory oversight to undergo plasma therapies. These procedures are often conducted without adequate clinical follow-up or emergency care infrastructure, further amplifying risks.

Perhaps most insidious is the spread of misinformation, particularly when fueled by statements or social media posts from high-profile scientists, investors, or celebrities. The public may not appreciate the distinction between mechanistic findings in mouse models and robust evidence of clinical efficacy in humans. This misinformation fosters unrealistic expectations, undermines public trust, and diverts attention from scientifically grounded gerotherapeutics.

The U.S. Food and Drug Administration addressed these concerns in a 2019 safety communication, warning against the use of young donor plasma for unapproved indications such as aging, memory loss, or neurodegenerative diseases [65]. The agency cited a lack of proven clinical benefit, potential for harm, and a concerning trend toward commercial overreach. In a 2024 update, the FDA reiterated its 2019 guidance [66], emphasizing that “young plasma” has not been approved for any anti-aging, cognitive, or neurodegenerative indications, and that no credible scientific evidence exists to support its efficacy in treating conditions such as dementia, Alzheimer’s disease, cardiovascular disease, or aging-related cognitive decline. The agency expressed specific concern about clinics charging thousands of dollars for infusions of plasma collected from donors aged 18–30 years, often under claims unsupported by regulatory approval or validated clinical evidence. It was emphasized that even if a study appears on clinicaltrials.gov or a clinic is FDA-registered, these factors do not imply FDA endorsement, approval, or evidence of safety and effectiveness. From a regulatory standpoint, plasma is currently approved only for clearly defined clinical scenarios, such as coagulation disorders, massive transfusions, and rare plasma protein deficiencies, as outlined in the FDA-recognized *Circular of Information for the Use of Human Blood and Blood Components* [67]. Its off-label use for “wellness” or anti-aging purposes falls outside any approved indication and FDA emphasizes the significant medical risks, including transmission of infectious diseases, allergic reactions, and respiratory complications. According to the FDA, any use of plasma exclusively collected from young donors outside of approved indications must occur under a formal Investigational New Drug application, overseen by qualified investigators. They further encourage clinicians and patients to report adverse events to the FDA’s MedWatch program, reinforcing the need for rigorous pharmacovigilance in this emerging and ethically sensitive domain. In sum, the FDA’s position underscores the clear disjunction between preclinical enthusiasm and clinical readiness, advocating for restraint, oversight, and adherence to evidence-based medicine in any future translation of plasma-based interventions.

## Plasma dilution therapies in humans

Plasma dilution approaches, particularly therapeutic plasma exchange, have gained considerable attention as potential strategies to modify the systemic environment and promote healthy aging. TPE in humans involves the removal of a portion of a patient’s plasma, which is then replaced with saline, albumin, or donor plasma to dilute circulating factors. This procedure, already well-established in clinical practice for certain autoimmune and hematological disorders, has recently been repurposed in experimental settings with the aim of attenuating age-related biological processes. Yet, several commercial ventures have positioned TPE as an emerging anti-aging therapy, often marketing it to the general public despite limited supporting evidence [61].

Interest in TPE as an anti-aging intervention has been fueled by preclinical studies in rodents, where dilution of plasma, without the infusion of young blood, was shown to reduce markers of biological aging, reduce systemic inflammation, improve tissue repair, and enhance function across multiple organ systems [4, 5]. These findings suggest that selectively removing or diluting circulating factors that accumulate with age may help mitigate aspects of age-related decline. However, it is important to acknowledge that even in animal studies, the precise mechanisms underlying these benefits remain incompletely understood. Moreover, concerns persist regarding the potential dilution or loss of essential anti-geronic components in the process, raising important questions about unintended consequences.

A deeper challenge lies in the inherent non-specificity of plasma dilution. Plasma is a remarkably complex and dynamic biofluid, containing thousands of proteins, lipids, metabolites, cytokines, antibodies, and extracellular vesicles [2]. Within this intricate mix reside both pro-geronic (aging-promoting) and anti-geronic (youth-promoting) factors. While the removal or dilution of toxic elements, such as senescence-associated secretory phenotype factors, pro-inflammatory cytokines, and certain microRNAs, might seem beneficial, the procedure also inadvertently reduces or eliminates critical homeostatic components. These include neurotrophic hormones, growth factors like IGF-1, essential nutrients, and immunoregulatory proteins that contribute to tissue repair, neurovascular integrity, and immune balance. As such, plasma dilution represents a blunt tool applied to a delicate physiological system. The key issue is not only whether plasma dilution offers measurable benefit, but also what is lost in the process. Without precise identification and targeting of deleterious factors, the risk of removing beneficial components may outweigh the theoretical advantages. Future strategies will need to evolve from indiscriminate dilution to selective modulation—leveraging insights from parabiosis and plasma studies to develop targeted biologics, neutralizing antibodies, or molecular mimetics that can recapitulate the positive effects of a youthful systemic milieu without undermining essential homeostatic balance. In this context, while plasma dilution has provided useful experimental insights, its translation into clinical practice must proceed with caution, scientific rigor, and humility. Responsible therapeutic development requires a far more nuanced understanding of the molecular architecture of plasma and the interplay between circulating factors before such interventions can be deemed safe, effective, or ethically sound for human aging.

In humans, robust evidence supporting the rejuvenative potential of TPE is limited [68, 69]. Early pilot studies have reported modest reductions in inflammatory markers such as C-reactive protein (CRP) and interleukin-6 (IL-6), alongside subjective improvements in fatigue and cognition in older adults. However, these studies were generally small, methodologically limited, lacked rigorous controls, and used heterogeneous protocols, making their interpretation challenging. Furthermore, findings have not been consistently replicated in larger, more diverse populations. A recent crossover study in healthy adults [70] assessed the effects of repeated plasmapheresis without replacement fluids on biomarkers of aging. While expected changes in lipids and electrolytes were observed, epigenetic clocks showed accelerated biological aging with more sessions, contradicting the notion of rejuvenation. These findings highlight that not all plasma-modifying interventions yield beneficial outcomes, and some may, in fact, impose physiological stress without evidence of anti-aging effects. A recent randomized, single-blinded, placebo-controlled study by Fuentealba and colleagues sought to provide more comprehensive insights into the biological effects of TPE in healthy older adults [68]. In this study, participants over the age of 50 were assigned to various TPE regimens or a placebo group, with the primary objective of assessing safety and evaluating biological age using a multi-omics framework. This approach incorporated a wide array of biological markers, including DNA methylation profiles, proteomic and metabolomic signatures, and immune system characteristics. The study reported that TPE was generally well tolerated, with a low incidence of adverse events. Changes were observed in several omics-based biomarkers, including reductions in certain epigenetic age estimators and shifts in inflammatory profiles. These findings were interpreted as preliminary evidence that TPE may influence systemic biology in ways consistent with reduced biological age. However, several important caveats warrant emphasis.

First, the study’s relatively small sample size and short duration limit both the statistical power and the ability to assess long-term safety or sustained effects. Second, although termed “placebo-controlled,” the trial design included only single-blinding, and the method of randomization raises concerns regarding potential bias. Third, the study focused on healthy older adults, limiting the generalizability of the findings to individuals with age-related diseases or more advanced biological aging.

An important interpretive challenge in recent plasma dilution studies relates to the use of epigenetic clocks as surrogate outcome measures. While DNA methylation–based aging metrics provide valuable insights into biological aging trajectories, their application as interventional endpoints remains limited by several factors. Epigenetic clocks are tissue-specific, sensitive to changes in cell-type composition, and may reflect transient physiological perturbations rather than durable rejuvenation. Moreover, reductions in epigenetic age do not necessarily translate into functional improvement or disease modification, particularly in complex, age-related pathologies such as neurodegeneration. As such, clock-based readouts should be interpreted as exploratory biomarkers rather than definitive evidence of systemic rejuvenation, and future trials will require integration with functional, clinical, and tissue-specific endpoints. A key methodological consideration is the reliance on peripheral blood mononuclear cells (PBMCs) for assessing epigenetic age. PBMCs, while easily accessible, represent a heterogeneous mixture of immune cells whose proportions can fluctuate with aging, inflammation, or physiological stress. TPE itself can induce transient changes in immune cell composition, potentially confounding interpretations of DNA methylation–based age estimates. Thus, observed reductions in epigenetic age may reflect shifts in cell populations rather than true rejuvenation of cellular function. Furthermore, PBMC-derived clocks may not accurately capture biological aging processes in critical tissues such as the brain, vasculature, or musculoskeletal system, which are central to age-related disease and decline.

Additional concerns regarding the reproducibility of epigenetic age estimates have emerged. Notably, the sham-treated control group in the Fuentealba study exhibited variability in epigenetic age over time, highlighting the technical and biological instability of short-term clock-based measurements in small cohorts. Without extended follow-up and validation in larger studies, it remains difficult to distinguish true intervention effects from natural fluctuations or technical variability.

Importantly, no meaningful clinical endpoints, such as improved cognitive performance and physical function or validated biomarkers of disease, were demonstrated in this or other TPE studies focused on aging. While the molecular findings are intriguing, their translational relevance remains speculative.

From a safety perspective, although TPE is widely used in clinical practice for well-defined indications, its application as a preventive or anti-aging intervention raises distinct challenges. Therapeutic plasma exchange and related procedures, such as plasmapheresis commonly performed in nephrology and hematology, are known to induce substantial physiological shifts. These procedures, which also dilute plasma and remove circulating factors, have well-documented side effects, including hypotension, electrolyte disturbances, coagulopathies, and increased susceptibility to infection—particularly in older or frail patients with limited physiological reserves [71–73]. Nephrologists, who routinely perform plasmapheresis in conditions such as Goodpasture syndrome, ANCA-associated vasculitis, or thrombotic thrombocytopenic purpura, are acutely aware of the procedure’s risks and the need for careful monitoring of fluid balance, calcium levels, and coagulation parameters during and after treatment.

Importantly, the established clinical use of plasmapheresis typically involves finite treatment courses targeted to acute or life-threatening conditions, not chronic or repeated interventions in otherwise healthy individuals. The extrapolation of these protocols to the context of aging raises additional concerns. The cumulative physiological burden of serial TPE sessions aimed at modifying aging trajectories is poorly understood, and the long-term impact on immune function, vascular integrity, and hemostasis remains unknown. Moreover, repeated plasma dilution may inadvertently reduce critical homeostatic components, including coagulation factors, immunoglobulins, and essential hormones, with unclear consequences in older adults.

The inclusion of Alzheimer’s disease in the 9th Edition of the American Society for Apheresis (ASFA) Guidelines as a Category III, Grade 2 indication provides a cautious acknowledgment by the apheresis community that therapeutic plasma exchange is a potential area for investigation in Alzheimer’s disease [42]. However, this classification also underscores that the evidence remains weak and inconclusive, and ASFA does not endorse TPE as a standard treatment for Alzheimer’s disease or aging. The persistent predominance of Category III/Grade 2 C classifications across many proposed TPE applications highlights the need for careful validation before widespread use. Consistent with the ASFA guidelines, clinical application of TPE for aging-related indications should follow rigorous evidence of efficacy and safety rather than mechanistic plausibility alone.

In summary, while TPE represents a scientifically interesting approach to modulating the systemic environment, existing human studies provide only preliminary and inconclusive evidence for its effectiveness as an anti-aging intervention. Several ongoing clinical trials aim to further explore the safety, biological effects, and potential anti-aging benefits of therapeutic plasma exchange. For example, NCT05004220 is a Phase 2 study evaluating the impact of TPE on epigenetic aging markers and inflammatory profiles in older adults. Similarly, NCT03353597 investigates how plasma exchange affects biomarkers associated with aging and cognitive function. Additional studies such as NCT05769575 and NCT04678079 are focused on assessing plasma dilution or exchange in the context of healthy longevity, including metabolic and cognitive endpoints. However, the biological mechanisms, optimal protocols, and long-term safety profile are not yet fully understood. While these trials reflect growing interest in modulating the systemic environment to influence aging, results are pending, and their designs vary widely in terms of intervention protocols, outcome measures, and sample sizes. Until conclusive evidence emerges, the clinical application of plasma-based rejuvenation strategies remains investigational. As with other plasma-based interventions, enthusiasm for TPE should be tempered by scientific caution, and its use outside rigorously designed clinical trials remains premature.

## Concluding perspectives

The translational promise of systemic rejuvenation lies not in the transfusion of complex biological fluids, but in the extraction of mechanistic insights from them. Parabiosis and plasma transfer models have profoundly shaped our understanding of how circulating factors influence aging in a cell-non-autonomous fashion. These experimental paradigms have revealed that the systemic environment exerts powerful regulatory control over cerebrovascular, immune, and neuronal aging. However, the leap from mechanistic discovery to clinical application is far from trivial, and the premature implementation of plasma-based therapies may ultimately be more harmful than helpful.

The indiscriminate transfer of young plasma or systemic factors is analogous to unregulated polypharmacy—akin to individuals taking dozens of supplements without understanding potential interactions or cumulative effects. This approach, exemplified by biohacking regimens like those of Bryan Johnson, poses substantial unknown risks, especially when manipulating growth factors, peptides, and hormones simultaneously. Of note, age-associated declines in certain circulating factors, including growth factors, may serve protective roles, particularly against aggressive cancers. For example, neoplasms in older individuals often exhibit a slower, indolent course. A youthful systemic environment, if introduced indiscriminately, may reawaken dormant tumor growth or promote metastatic potential.

Rather than embracing plasma itself as a therapy, the future of geroscience must focus on precisely targeted interventions informed by plasma research. These include the development of recombinant forms or molecular mimetics of identified anti-geronic molecules, as well as small-molecule inhibitors or antibodies that selectively neutralize pro-aging cytokines and other deleterious circulating factors. Equally important are biomarker-guided strategies—leveraging advances in proteomics, metabolomics, and epigenetics—to enable patient stratification, individualized treatment, and rigorous monitoring of therapeutic responses.

In parallel, robust regulatory frameworks are essential to safeguard the safety, efficacy, and ethical integrity of emerging rejuvenation interventions. Any therapy claiming to slow or reverse aging must undergo methodologically sound, randomized clinical trials with long-term follow-up, appropriate controls, and validated outcome measures. Commercial enthusiasm, media attention, or preclinical excitement cannot substitute for scientific rigor.

As we stand at the intersection of groundbreaking discovery and translational ambition, the path forward must be anchored in evidence, mechanistic clarity, and ethical responsibility. Responsible geroscience demands not haste, but humility—a commitment to doing what works, not merely what excites.
